# Impact of conflict on maternal and child health service delivery: a country case study of Afghanistan

**DOI:** 10.1186/s13031-020-00285-x

**Published:** 2020-06-10

**Authors:** Shafiq Mirzazada, Zahra Ali Padhani, Sultana Jabeen, Malika Fatima, Arjumand Rizvi, Uzair Ansari, Jai K. Das, Zulfiqar A. Bhutta

**Affiliations:** 1 Academic Projects Afghanistan, Aga Khan University, Kabul, Afghanistan; 2 Division of Women and Child Health, Aga Khan University, Karachi, Pakistan; 3 Department of Paediatrics and Child Health, Aga Khan University, Karachi, Pakistan; 4 Center of Excellence in Women and Child Health, Aga Khan University, Karachi, Pakistan; 5 Centre for Global Child Health, The Hospital for Sick Children, 686 Bay Street, Toronto, ON M5G 0A4 Canada

**Keywords:** Conflict, Humanitarian, Afghanistan, Maternal health, Child health, Nutrition

## Abstract

**Introduction:**

Since decades, the health system of Afghanistan has been in disarray due to ongoing conflict. We aimed to explore the direct effects of conflict on provision of reproductive, maternal, newborn, child and adolescent health and nutrition (RMNCAH&N) services and describe the contextual factors influencing these services.

**Method:**

We conducted a quantitative analysis of secondary data on RMNCAH&N indicators and undertook a supportive qualitative study to help understand processes and contextual factors. For quantitative analysis, we stratified the various provinces of Afghanistan into minimal-, moderate- and severe conflict categories based on battle-related deaths from Uppsala Conflict Data Program (UCDP) and through accessibility of health services using a Delphi methodology. The coverage of RMNCAH&N indicators across the continuum of care were extracted from the Demographic and Health Surveys (DHS) and Multiple Indicator Cluster Survey (MICS). The qualitative data was captured by conducting key informant interviews of multi-sectoral stakeholders working in government, NGOs and UN agencies.

**Results:**

Comparison of various provinces based on the severity of conflict through Delphi process showed that the mean coverage of various RMNCAH&N indicators including antenatal care (OR: 0.42, 95%CI: 0.32–0.55), facility delivery (OR: 0.42, 95%CI: 0.32–0.56), skilled birth attendance (OR: 0.43, 95%CI: 0.33–0.57), DPT3 (OR: 0.26, 95% CI: 0.20–0.33) and oral rehydration therapy (OR: 0.37, 95% CI: 0.25–0.55) was significantly lower for severe conflict provinces when compared to minimal conflict provinces. The qualitative analysis identified various factors affecting decision making and service delivery including insecurity, cultural norms, unavailability of workforce, poor monitoring, lack of funds and inconsistent supplies. Other factors include weak stewardship, capacity gap at the central level and poor coordination at national, regional and district level.

**Conclusion:**

RMNCAH&N service delivery has been significantly hampered by conflict in Afghanistan over the last several years. This has been further compromised by poor infrastructure, weak stewardship and poor capacity and collaboration at all levels. With the potential of peace and conflict resolution in Afghanistan, we would underscore the importance of continued oversight and integrated implementation of sustainable, grass root RMNCAH&N services with a focus on reaching the most marginalized.

## Background

Afghanistan is a landlocked country located between the Middle East, Central and South Asia, with 34 provinces and an estimated population of 31,575,018 [1]. The health services of Afghanistan are overseen by the Ministry of Public Health (MoPH) which caters to the health needs of the country, which has been in the throes of conflict for well over four decades [2]. Conflict in Afghanistan has severely compromised the provision of services by the public and private sectors in areas of economics, health, education, livelihood, agriculture and livestock amongst others. Afghan conflict started after the invasion by the Soviet Union in 1979, followed by an uprising of the Mujahedeen (guerrilla-type militant groups led by the Islamist Afghan fighters). Following the defeat and withdrawal of the Soviet forces and a period of anarchy, the Taliban movement started with an aim to end the civil war and establish Sharia law in the country and succeeded to overthrow the Mujahedeen government by end of 1990s. However, the next decade of rule by the Taliban also witnessed tremendous reduction in primary care services, oppression and marginalization of women and growth of radicalism. The Taliban government was overthrown by the US and the coalition partners after the 9/11 attack on World Trade Center, who then withdrew to rural districts and tribal regions in the Pakistan-Afghanistan border. This civil war continues to date with varying intensity and a peace agreement was recently signed but with uncertain consequences.

Although quantitative data from population surveys in the decade prior to the overthrow of the Taliban is negligible, the health system was in total disarray due to the long ensuing fighting and poor governance. The maternal and child mortality rates were amongst the worst across the globe and the coverage of reproductive, maternal, newborn, child and adolescent health and nutrition (RMNCAH&N) interventions were very low.

The MOPH with the support of non-governmental organizations (NGOs) and international donors introduced the Basic Package of Health Services (BPHS) in 2003 which provided strategic primary healthcare services. This was upgraded in 2005 to additional hospital services of Essential Package of Hospital Services (EPHS). With the implementation of BPHS and EPHS, the health indicators improved; infant and under five morality dropped to 45 and 55 per 1000 live births respectively and maternal mortality ratio was reported at 327 in the Afghanistan Mortality Survey in 2010 [3]. The health infrastructure also improved in terms of quantity and quality of services provided.

**Fig. 1:**
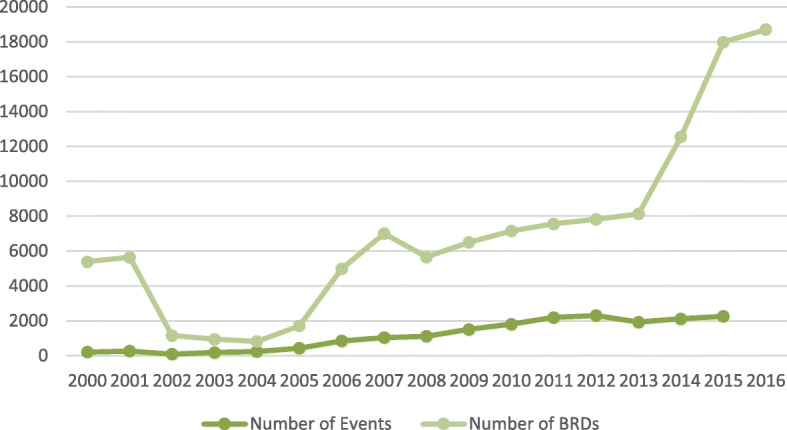
Conflict related events and battle related deaths in Afghanistan

The study presented here is part of a larger, multi-country study undertaken by the Bridging Research and Action in reproductive, maternal, newborn, child, and adolescent health (BRANCH) Consortium focusing on ten conflict-affected countries including Afghanistan, Colombia, DRC, Mali, Nigeria, Pakistan, Somalia, South Sudan, Syria, Yemen [4]. This country case study focuses on the period of the relatively recent conflict in Afghanistan, starting after the year 2001, and continuing to date. The severity and burden of conflict was measured from the metric of Battle Related Deaths (BRDs) and incidents according to Uppsala Conflict Data Program (UCDP) [5] (Fig. 1).

The aim of this study was to explore the impact of conflict on provision of RMNCAH&N services in Afghanistan, its progress and the factors that influence decision making and implementation of these services.

## Methods

### Study design

We conducted a quantitative analysis of secondary data to explore the impact of conflict on RMNCAH&N services and a primary qualitative study to help understand the processes and contextual factors affecting the service delivery in conflict settings.

### Quantitative assessment methodology

#### Data sources

For coverage data on key RMNCAH&N indicators, we extracted data from nationwide household surveys including Multiple Indicator Cluster Survey (MICS) (2003, 2010) and Demographic and Health Survey (DHS) 2015 [6–8]. These surveys were used because of their standardized methodology, reliability and consistent definition of indicators. We extracted conflict data, including information on type of conflict, number of events and fatalities, location, and date of event from UCDP [5].

#### Conflict categorization

We categorized the provinces of Afghanistan into three groups – minimal, moderate and severe conflict. These provinces were stratified by using two different approaches. First, on the basis of number of battle related deaths (BRDs) from the UCDP (Minimal: < 100 BRDs, Moderate: 100–300 BRDs, Severe: > 300 BRD) and second on the basis of Delphi exercise which classified these areas on the basis of perceived insecurity by stakeholders. The reason why we have used both measures is the often the noted lack of link between insecurity and access issues and location of BRDs [9], as many terrorism related events (such as car bombs and mass attacks) are disproportionately clustered in large urban centers like Kabul and provincial capitals with considerably more service providers and facilities.

The Delphi technique [10–12] is a well-established instrument to reach consensus from a panel of experts on questions that cannot be easily answered with empirical evidence and complete certainty [13]. We identified 41 key stakeholders involved in the planning and implementation of health service delivery at the central and provincial level in Afghanistan representing the government, UN agencies, NGOs and academia through a purposive sampling strategy. Reasonably diverse and representative geographic representation was sought specifically in order to ensure generalizability. The expert panel was provided with a Likert scale rating questionnaire by email and each of the experts rated the provinces as mild, moderate- and severe- conflict separately for the period 2003–2010 and 2010–2018 based broadly on the accessibility to health services for the health-care providers and the community. Non-responders received reminder emails after 2 and 4 days, and were excluded if no response was obtained thereafter. The votes of all experts were weighed equally within the Delphi process and provinces of Afghanistan were then grouped into low-, moderate- and high intensity conflict groups when more than 70% of the experts had classified them to a particular conflict category for the two time periods separately.

#### Quantitative data analysis

We performed stratified descriptive analyses, and calculated means/standard deviations, and frequencies/proportions for the coverage of RMNCAH&N indicators from MICS and DHS based on the severity of conflict. Coverage of key RMNCAH&N services indicators were extracted which included contraceptive use, antenatal care (ANC), tetanus toxoid (TT), skilled birth attendance (SBAs), facility birth, immunization (BCG, DPT3/Penta3), early initiation of breastfeeding, exclusive breastfeeding (EBF), continued breastfeeding till 2 years of age, vitamin A supplementation, care-seeking for acute respiratory infections (ARI) and use of oral rehydration therapy. The Composite Coverage Index (CCI) [14] was calculated as a weighted coverage mean of eight select RMNCAH&N interventions along the continuum of care. These interventions included family planning services (FPS), ANC, SBA, immunizations (BCG, DPT3, and measles), oral rehydration therapy (ORT) and case management of ARI.

$$ CCI=\raisebox{1ex}{$1$}\!\left/ \!\raisebox{-1ex}{$4$}\right.\left( FPS+\frac{SBA+ ANCS}{2}+\frac{2 DPT3+ MSL+ BCG}{4}+\frac{ORT+ CPNM}{2}\right) $$

Each continuum stage was given equal weight (Note that FPS indicates family planning needs satisfied (related to contraceptive use) and CPNM refers to care seeking for ARI]

The net mean and mean differences of coverage estimates of the various indicators from 2003 to 2015 were compared across conflict subgroups (minimal, moderate and severe) using the Student’s t-tests and one-way analysis of variance methods. Post-hoc comparisons were conducted with the Tukey’s multiple comparison methods. We then examined the impact of conflict on coverage indicators using binary logistic regression approach. The primary exposure was conflict status and the analysis was adjusted for time, place of residence, maternal education and wealth status. In addition, the analysis was adjusted for survey design and sampling weights through ‘svy’ routine in STATA version 15 [15]. Bivariate analysis was conducted to evaluate the effect of primary exposures on outcomes and the multivariate adjusted models were developed with time with and without covariates to assess the impact of the primary exposure with time on the RMNCAH&N outcomes. The adjusted estimates were reported as odds ratios (OR) with 95% confidence intervals (CI) and the *p*-value less than 0.05 was considered significant.

### Qualitative assessment methodology

We undertook primary qualitative data collection through key informant interviews with stakeholders from Government, development partners and NGOs identified through a purposive sampling method and snowball technique (Table 2). The provinces and the stakeholders were decided after consultation with the members of the BRANCH consortium and stakeholders at the central level in Kabul including government, development partners, NGOs and academia. To capture varied perceptions; we selected stakeholders with different background and varied experiences of policy making, implementation and monitoring and evaluation focusing on those with experience at different stages over the last decade. Given security concerns about movement and sampling from all provinces, we chose representatives from the national level based at Kabul and from the provinces of Faryab and Helmand to get deeper insights to the contexts and these two provinces were selected by consensus given the two major ethnic and geographic diversities. All interviews were based on a semi-structured interview guide for each targeted group. Interviews lasted approximately for an hour and were conducted in English which were audio recorded and then transcribed. Latent content analysis was used to analyze the data and thematic analysis (combining an inductive and deductive approach) was used with the assistance of NVivo version 10.0 software [15].

## Results

**Fig. 2:**
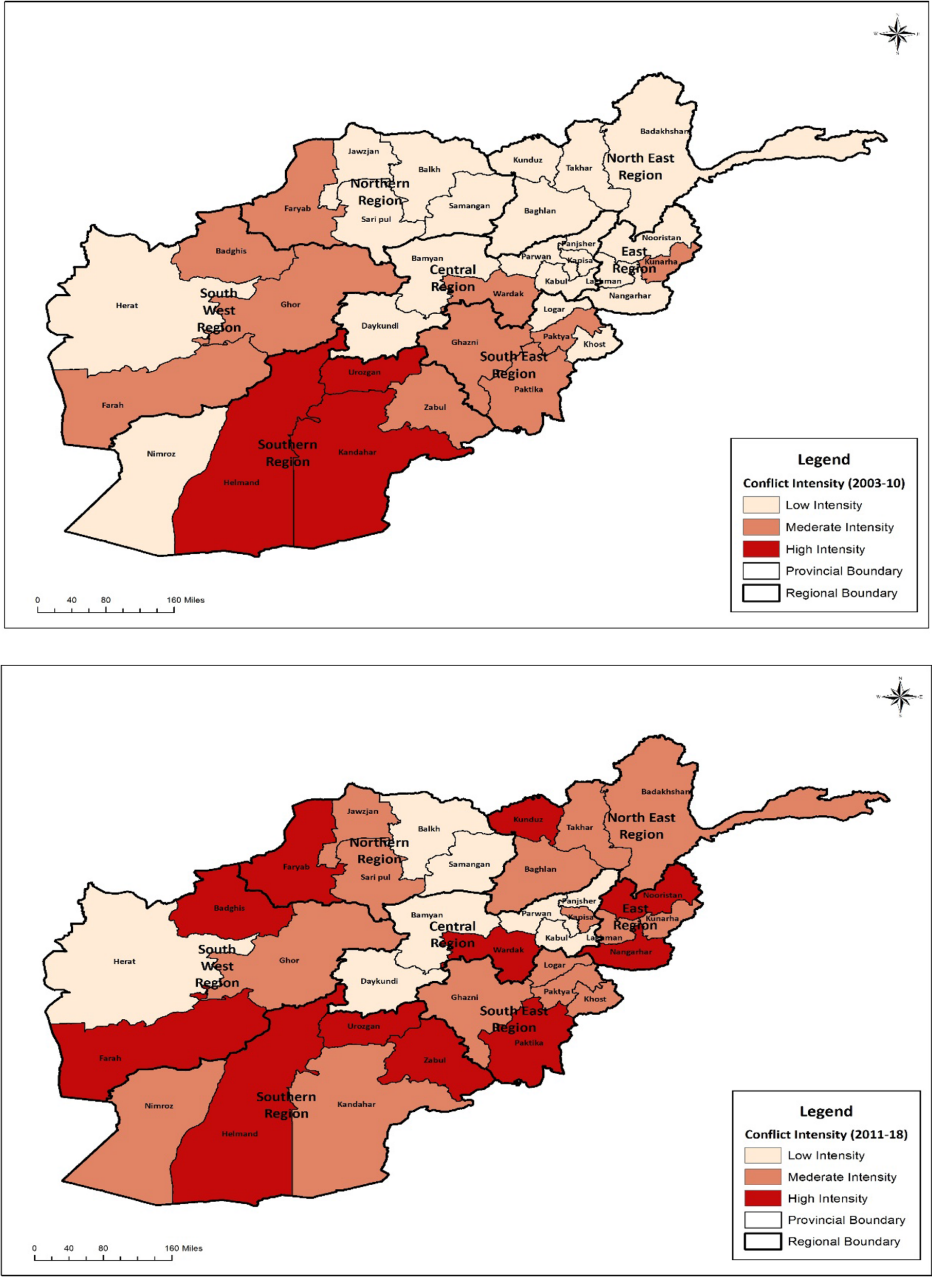
Provincial ratings by conflict severity in 2003–2010 and 2010–2018 based on the Delphi exercise

**Fig. 3:**
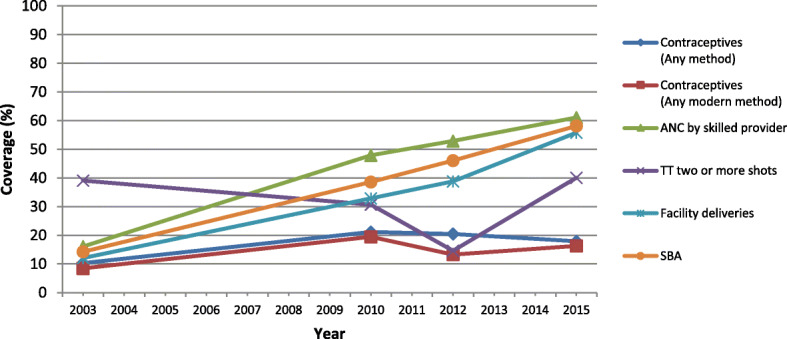
National trends in reproductive and maternal interventions from 2003 to 2015

**Fig. 4:**
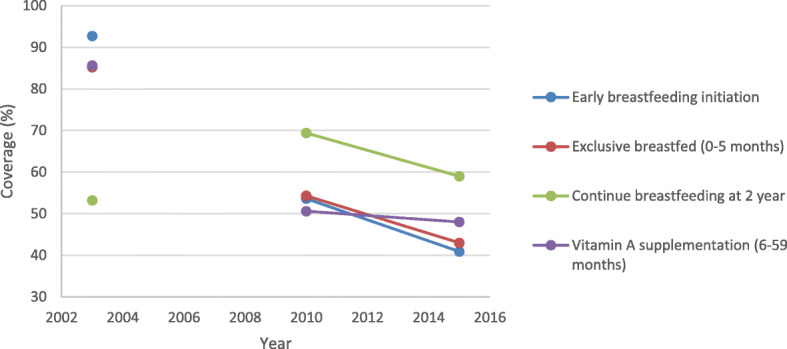
National trends in nutritional interventions from 2002 to 2016

Associations between Conflict Severity and Intervention Coverage: Fig. 2 shows the ratings of the provinces based on the conflict severity through the Delphi methodology for the years 2003–2010 and 2010–2018. The trends in the coverage of key RMNCAH&N service indicators suggest that despite being in the midst of conflict, Afghanistan has made progress in improving the coverage of RMNCAH&N service coverage indicators and most of the indicators examined show improvements across the 2003–2015 period. The coverage of ANC and measles immunizations increased at a faster pace in 2003–2010 relative to 2010–2015 and the TT coverage, facility deliveries, SBA, BCG and DPT3/ Penta3 coverage as well as the CCI increased at a faster pace during 2010–2015 (Fig. 3). Coverage of contraceptives increased until 2010, but declined slightly till 2015. The coverage data of several nutrition indicators including early initiation of breastfeeding, exclusive breastfeeding, continued breastfeeding at 2 years and vitamin A supplementation were unreliable in the 2002 surveys, but subsequent surveys have shown reduction in the coverage of most nutrition indicators after the year 2010 (Fig. 4). But even with some progress, there are notable disparities between provinces.

**Fig. 5:**
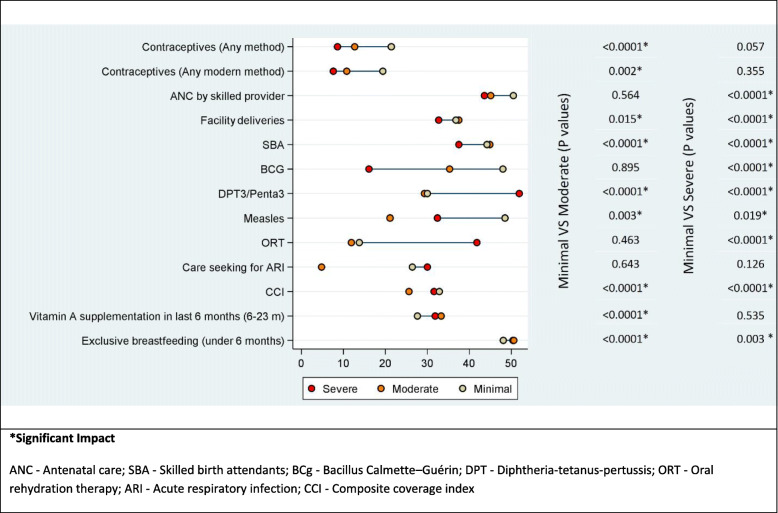
Mean difference in the coverage of key RMNCH indicators for Afghanistan by conflict category based on the Delphi methodology from 2003 to 2015

Impact of Conflict: The provinces were rated as in mild-, moderate- and severe conflict for the period of 2003–2010 and 2010–2018 based on both the BRD and the Delphi exercise (Fig. 2). The bivariate analysis based on the BRD classification showed no statistical significant differences in the coverage of RMNCAH&N services between the severe and moderate conflict-affected provinces when compared to mild conflict provinces. The classification using the Delphi methodology shows that the severity of conflict is associated with the health service delivery as the mean difference in the coverage of RMNCAH&N services for the period 2003–2015 was significantly lower for moderate and severely conflict-affected provinces when compared to those minimally affected across a range of indicators (Fig. 5). The multivariate analysis also showed that the coverage for indicators including ANC (OR: 0.42, 95%CI: 0.32–0.55), facility delivery (OR: 0.42, 95%CI: 0.32–0.56), SBA (OR: 0.43, 95%CI: 0.33–0.57), DPT3 (OR: 0.26, 95% CI: 0.20–0.33) and ORT (OR: 0.37, 95% CI: 0.25–0.55) was significantly lower for severe conflict provinces when compared to the minimal conflict provinces. The mean difference in the coverage of EBF under 6 months (OR: 4.44, 95% CI: 1.86–10.61) and BCG (OR: 2.80, 95% CI: 2.09–3.76) was significantly higher in severe conflict compared to the minimal conflict provinces. There was no significant difference in the coverage of contraceptive - any method (OR: 0.98, 95% CI: 0.74–1.30), contraceptive – any modern method (OR: 1.12, 95% CI: 0.83–1.30) and care-seeking for ARI (OR: 0.73, 95%CI: 0.49–1.09). The moderate conflict provinces had a significantly lower coverage of contraceptive- any method (OR: 0.77, 95% CI: 0.66–0.90), contraceptive – any modern method (OR: 0.83, 95% CI: 0.71–0.96), facility delivery (OR: 0.75, 95%CI: 0.58–0.96), BCG (OR: 0.75, 95%CI: 0.58–0.98), DPT/Penta 3 (OR: 0.70, 95%CI: 0.54–0.91), vitamin A supplementation (OR: 0.58, 95% CI: 0.45–0.75), when compared to the minimal conflict provinces (Table 1).

**Table 1:** Multivariate analysis for Afghanistan for the effect of conflict on key indicators using the Delphi methodology

Outcome	Conflict Type	Bivariate				Multivariate			
		OR.	95% CI		***p***-value	OR.	95% CI		***p***-value
**Contraceptive any method**	Minimal	Ref.				Ref.			
	Moderate	0.73	0.63	0.84	< 0.0001	0.77	0.66	0.90	0.001
	Severe	0.76	0.57	1.01	0.057	0.98	0.74	1.30	0.896
**Contraceptive any modern method**	Minimal	Ref.				Ref.			
	Moderate	0.81	0.70	0.93	0.002	0.83	0.71	0.96	0.014
	Severe	0.87	0.65	1.17	0.355	1.12	0.83	1.51	0.450
**ANC by skilled provider**	Minimal	Ref.				Ref.			
	Moderate	0.94	0.77	1.15	0.564	1.06	0.86	1.31	0.564
	Severe	0.28	0.22	0.36	< 0.0001	0.42	0.32	0.55	< 0.0001
**SBA**	Minimal	Ref.				Ref.			
	Moderate	0.78	0.64	0.95	0.015	0.88	0.71	1.10	0.268
	Severe	0.29	0.22	0.37	< 0.0001	0.43	0.33	0.57	< 0.0001
**Facility Birth**	Minimal	Ref.				Ref.			
	Moderate	0.65	0.52	0.81	< 0.0001	0.75	0.58	0.96	0.025
	Severe	0.27	0.21	0.36	< 0.0001	0.42	0.32	0.56	< 0.0001
**BCG**	Minimal	Ref.				Ref.			
	Moderate	1.02	0.78	1.34	0.895	0.75	0.58	0.98	0.036
	Severe	3.12	2.38	4.09	< 0.0001	2.80	2.09	3.76	< 0.0001
**DPT3/PENTA3**	Minimal	Ref.				Ref.			
	Moderate	0.47	0.38	0.58	< 0.0001	0.70	0.54	0.91	0.007
	Severe	0.25	0.19	0.32	< 0.0001	0.26	0.20	0.33	< 0.0001
**Measles**	Minimal	Ref.				Ref.			
	Moderate	1.49	1.15	1.93	0.003	1.21	0.91	1.61	0.194
	Severe	1.52	1.07	2.16	0.019	1.36	0.95	1.94	0.096
**ORS**	Minimal	Ref.				Ref.			
	Moderate	0.91	0.70	1.17	0.463	1.28	0.95	1.72	0.099
	Severe	0.32	0.21	0.48	< 0.0001	0.37	0.25	0.55	< 0.0001
**Care seeking for ARI**	Minimal	Ref.				Ref.			
	Moderate	1.06	0.82	1.38	0.643	1.09	0.84	1.43	0.504
	Severe	0.73	0.49	1.09	0.126	0.73	0.49	1.09	0.122
**EBF under 6 months**	Minimal	Ref.				Ref.			
	Moderate	1.82	1.32	2.52	< 0.0001	2.59	1.79	3.73	< 0.0001
	Severe	2.91	1.56	5.43	0.003	4.44	1.86	10.61	0.001
**Vitamin A in past 6 months**	Minimal	Ref.				Ref.			
	Moderate	0.52	0.41	0.67	< 0.0001	0.58	0.45	0.75	< 0.0001
	Severe	0.89	0.62	1.28	0.53	0.94	0.65	1.36	0.756

*ANC* Antenatal care, *SBA* Skilled birth attendants, *BCG* Bacillus Calmette–Guérin, *DPT* Diphtheria-tetanus-pertussis, *ORT* Oral rehydration therapy, *ARI* Acute respiratory infection, *CI* Confidence interval, *EBF* Exclusive Breastfeeding

### Contextual factors

**Fig. 6:**
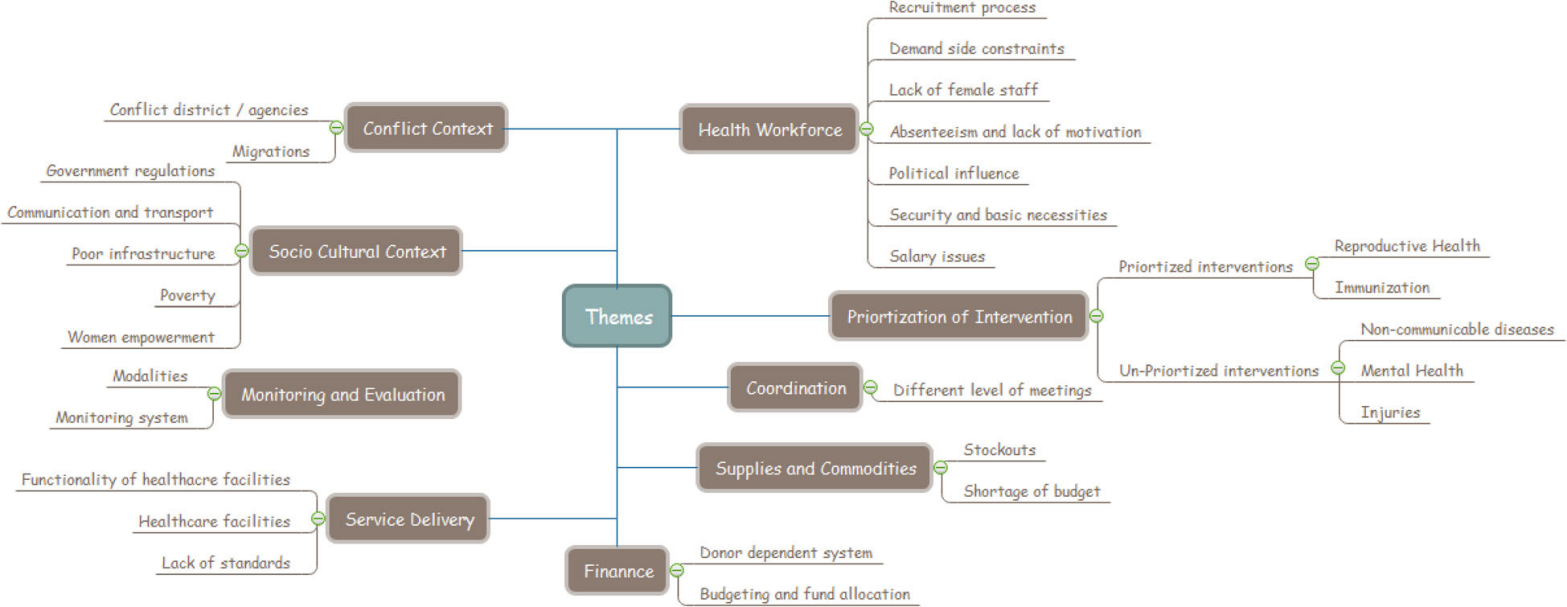
Themes from Key Informant Interviews

A total of 34 interviews were conducted and the distribution and the demographics of the stakeholders is detailed in Table 2. Following major themes were identified through the key informant interviews and the findings are based on the opinions for most of the stakeholders interviewed (Fig. 6).

**Table 2:** Participant Demographics of Key Informant Interviews

Variable	Key Informant Interviews	
	No.	(%)
**Sample size**		
Number of participants	34	
**Location**		
Faryab	13	(38.2)
Helmand	13	(38.2)
Kabul	08	(23.5)
**Sex**		
Male	11	(32.3)
Female	23	(67.6)
**Education**		
Secondary Education	00	(00)
Graduate	25	(73.5)
Masters or Other Advanced Degree	09	(26.4)
**Experience, years**		
Below 10	11	(32.3)
10–20	18	(52.9)
21–30	03	(8.8)
30 Above	02	(5.8)
**Organization**		
Development Partners	05	(14.7)
Government	13	(38.2)
NGOs	16	(47.0)
**Type of staff**		
Center Based	27	(79.4)
District Based	00	(00)
Facility Based	07	(20.5)

#### Conflict

Since the past four decades, influence of Taliban and anti-government elements has severely affected the healthcare service delivery in Afghanistan. There were still many areas which were in control of Taliban and some provinces are in grip of continuous war and these areas were the most hard to access and lacked provision of services. These uncertain security conditions had forced many people to move to the adjacent districts (Internally Displaced People (IDPs)) and has disrupted their livelihood, economic status and health.

*“Economic situation of people has worsened in war and displacement of people has led to increased incidents of malnutrition in mothers and children, which is another challenge.” [KII-13-NGO-Faryab]*

#### Infrastructure and socio-cultural

Afghanistan has faced numerous challenges related to poor infrastructure and economic distress, as it has been in a state of war for a long time. The poor infrastructure including poor road network, lack of transport, difficult terrain and far away health facilities made health care delivery difficult and inaccessible for people especially living in rural areas. Afghanistan is a male dominant conservative society with high rate of illiteracy, which poses serious barriers to the free movement of women. Some tribes in Afghanistan still prefer being treated by traditional or religious healers rather than a healthcare professional.

*“Some religious people misinterpreted Islam and they thought that women have no right to leave her house. She was also not allowed to seek education or work in a health facility.” - [KII-3-UN-Ka]*

#### Prioritizing of interventions

The priority of health service delivery had been on RMNCH&N services through BPHS and EPHS with a specific focus on ANC, care at birth and postnatal care (PNC) and immunization (specifically polio) and a low focus on Expanded Program on Immunization (EPI). The emphasis on increasing awareness and practices for breastfeeding and other preventive measures was lacking and it was suggested to initiate programs solely focusing on improving infant and young child feeding (IYCF) practices in order to prevent undernutrition and reduce childhood morbidity and mortality. The BPHS and EPHS are not fully catering to the health needs of the Afghan population, as there was less or no priority given to important areas including non-communicable diseases, mental health, injuries which share the major burden of disease. The specific focus has also been on the primary healthcare model of BPHS and this has affected tertiary and specialized quality care.

Apart from BPHS, other healthcare programs including vertical programs for specific diseases relied solely on the available funding and particular interest of donors and hence were not sustainable once the funding subsided. The other challenges included weak coordination and interference of political and community leaders.

*“Weak coordination at different levels, negative competitions, political and local elders’ interference in the program implementation have a negative impact on the prioritization of interventions” [KII-10-NGO-Faryab]*

#### Service delivery

In the model of BPHS, the service delivery is highly reliant on NGOs with minimum inputs from the existing technical departments within the MoPH, though these technical departments have the required capacity and strong association with development partners. The number of health care facilities are not adequate and are in poor state. Many primary healthcare facilities were non-functional due to insecurity, lack of infrastructure, shortages of staff, severe weather, migrations and poor patient flow. There is a need for regular maintenance and up-gradation of these health facilities for which a specific budget should be in place, together with a mechanism of monitoring. The secondary and tertiary healthcare units were also facing difficulties even in provision of basic care and most of these facilities lacked HIV prevention programs, orthopedic care, dental care, laboratory and screening tests and emergency care.

“The *major problem in Faryab province is that we don’t have an emergency hospital for children and for women, we also don’t have facility for STDs, especially for HIV. We also lack services for the management of unintended pregnancies and safe abortion” [KII-1-Gov-Faryab]*

#### Health workforce

The unpredictable security situation, threats, kidnapping and widespread trade of narcotics had left many people deprived of education and prevented people from working. Low salary packages and no facility of accommodation has also refrained experienced health professionals to work in peripheries and hence local hiring was preferred.

*“We had a lot of causalities while working here in Helmand. We lost a lot of our field workers during these years. We lost vaccinators, supervisors and coordinators.” [KII-10-UN-Helmand]*

The societal norms and restrictions imposed on women by Taliban had left most of the women uneducated over the years and together with the ongoing conflict had led to major deficiency of female doctors, midwives or nurses. NGOs working in the northern parts of the country in the past used to hire female doctors from neighboring countries like Tajikistan and Uzbekistan, as they shared the same language and culture and these workers were provided attractive salary packages, accommodation and transport. But with escalating conflict and unattractive salary packages with rising inflation and depreciation of Afghanistan currency, these foreign workers are no more attracted to work in Afghanistan. The community midwifery program which began training rural midwives in 2002 and scaling-up nationally in 2005 is largely seen as a successful program, which to a certain extent had been able to provide healthcare services to women in these difficult areas.

*“There are some socio-cultural and religious issues. Clients will not attend the health facilities if there are no female staff” [KII-7-NGO-Ka]*

The recruitment process was a lengthy and tedious process and not always transparent. The government hiring’s had to follow the national standard salary policy which were not competitive enough to attract qualified and competent individuals, as they were lured by the NGOs and the private sector through better packages. Hence the reliance has been to hire local health workers to overcome the shortcomings in resource constrained settings.

*“The recruitment process is very difficult and everyone knows this as a fact, but we try our best to recruit the staff through a recruitment committee, and we try to find qualified staff” [KII-6-UN-Helmand]*

In terms of development of human resources, there is no proper coordination between MoPH and the ministry of higher education and cross-sectional and interlinked coordination can promote public health education by improving ethics and capability in healthcare professionals.

#### Supplies and commodities

Government and NGOs were responsible to assure continuous flow of medicine and supplies, but this was a serious concern. The health facilities faced shortages of supplies due to insufficient budget allocation and security situation affecting delivery. Most of the local market suppliers were not equipped to meet the government regulatory requirements, hence a limited number of suppliers were available which were not enough to meet demands of all the 34 provinces. On the contrary, some participants mentioned that there were no shortages of medicines and were satisfied with the availability of supplies and commodities.

*“We had multiple developmental projects, but due to unavailability of budget and commodities, we were unable to start these projects and some even got delayed for years” [KII-1 Gov-Faryab]*

#### Finances

Afghanistan is a donor dependent country with a major chunk of these funds diverted towards war and security. The ministry has been receiving funds from World Bank (WB), European Union (EU), and United States Agency for International Development (USAID) for the successful implementation of BPHS services and also from GAVI for EPI. The budget allocation in Afghanistan takes place at the central level but lack of capacity, inadequate fund distribution, delay in release of funds and improper utilization poses major barriers to the successful implementation of the programs. This was a common realization that the donor support would end soon and government would then face an uphill task in maintaining even the current healthcare services. Hence it was urged that the government should soon explore alternate ways and finding solutions for the financing of the health system.

*“The achievements which we had in the past or even we have now is the result and outcome of our joint force and ownership and the funding from our donors.” – (Govt. official)*

#### Coordination

The coordination between various sectors is crucial for the efficient healthcare service delivery. Though there were coordination mechanisms in place at the central and provincial level including the Provincial Health Coordination Committee (PHCC), Community Based Health Care (CBHC), but these are not fully utilized as these committees did not regularly meet and there are no proper follow ups to monitor the progress. This apart from affecting healthcare delivery also sometimes led to duplication of effort.

#### Monitoring and evaluation (M&E)

There was no proper M&E system of Afghanistan, but there has been progress with introduction of regular checks and third party evaluations. There are now Standard Operating Procedures for the evaluation of NGOs performances. Technology has also been introduced with an upgraded digital EHIS system linking all the facilities. But there are still issues with the capacity and monitoring, as majority of the EHIS data was assumed to be poor quality and unreliable. The lack of capacity also hampered the regular and proper analysis and interpretation of data for reporting and decision making. Security was identified as the major bottleneck in monitoring and evaluation, as the supervisors were not able to make frequent visits to the monitoring sites.

## Discussion

Afghanistan has seen nothing but civil unrest and debilitating conflict since the past four decades which though targeting the Soviet invaders initially, has assumed ethnic dimensions. There is also the well-known engagement of several neighboring states and the USA given strategic interests; however most of the war weary population wants peace and there is hope that this might be in the offing. Clearly the dividends that peace may bring will be huge although the impoverished country may once again lose global interest and attention.

Regardless, Afghanistan is a case study in resilience [14] and our analysis shows the gains made by indigenous innovations [2]. The investments made early on in establishing core services such as the BPHS and EPHS and run by NGOs (both contracting in and out models) has ensured availability of health services in both rural and urban areas of the country. It has also enabled the provision of health services to the most insecure and remote areas of the country and assisted in scaling up RMNCAH&N services overall. The qualitative research findings of this study complement the quantitative findings and explores the contextual factors on the progress or lack thereof.

The quantitative analysis shows that even during conflict, Afghanistan has shown some progress as there has been an increase in coverage of most RMNCAH services including ANC, facility delivery, SBA, and vaccinations (BCG, DPT3 and TT coverage). The progress for a few indicators including breastfeeding, vitamin A supplementation and contraceptive use has declined after the year 2010 and this could be attributed to escalating conflict, donor dependence and poor capacity of healthcare system. The stagnant coverage of the use of contraceptives can also be linked to cultural norms, insecurity, illiteracy among women and socio-economic status of the people. These findings are not surprising as other studies have also reported on stagnant contraception rate and decreasing ANC and vaccination rates [2, 16]. A study reported low coverage of RMNCAH&N services among less wealthy households and stated that only half of the women were benefiting from it [2]. The study also showed that conflict significantly impacted health service delivery, as the coverage of most RMNCAH&N services through the continuum of care was significantly lower in provinces which had ongoing conflict and insecurity compared to provinces which were relatively free of conflict. Our study also highlights that BRDs may be a limited measure in assessing the severity and implications of conflict and other factors would need to be considered in determining ease of access to health services.

The qualitative analysis also highlights several interconnected contextual factors that affect the delivery of care and these are related to security, governance, service delivery, health workforce, finance, information system, supplies and monitoring (Table 3). Fear and insecurity has limited the mobility of health work forces and health care delivery is a great challenge. The notable gains in the healthcare indicators of Afghanistan has been severely compromised by the escalating insecurity which has plateaued the progress as also noted by other studies [16]. There should be a greater emphasis on prevention strategies especially on breastfeeding, immunizations and water, sanitation and hygiene. The current model of health service delivery also needs to be updated to bring in more focus to additional important services like mental health, orthopedics, injury and emergency care. Development in Afghanistan has been complicated by poor economy, unstable political system, fragmented healthcare system and poor baseline health indicators accompanied by ongoing conflict [17, 18]. A study also suggested that the government should work on strong monitoring and quality check authorities to end up corruption and improve healthcare [19].

**Table 3:** Facilitators, barriers and recommendation affecting health system in conflict areas of Afghanistan

	Facilitators	Barriers	Recommendations
Health Workforce	- Hiring of qualified local people along with incentives for retention	- Unpredictable security conditions	- Hire more female staff and reduce gender imbalance
		- Security threats	- Send female staff on rotation basis to conflict areas
		- Lack of female health workers	- Provide adequate training
		- Absenteeism and lack of capacity of healthcare staff	- Provide housing and basic necessities
		- - Low salaries	- Merit based hiring
		- Absence of accommodation and basic facilities for doctors	- Doctors or staff to provide replacements when going on leave
		- Quacks (traditional or religious healers) are preferred by people over doctors	
Service Delivery	- BPHS and EPHS has improved service delivery (through contracting out)	- Non-functional healthcare facilities	- SOPs should be implemented
		- Changing demographic pattern	- Work on infrastructure for the uptake of health care intervention
	- Establishment of various new primary healthcare facilities	- No services in remote areas	
		- Lack of HIV prevention program, orthopedic care, dental care, laboratory and screening tests and emergency care	- Stringent monitoring mechanisms using technology
			- Improve community awareness and mobilization activities
		- Poor infrastructure	- Improving CMWs functionality
Supplies and Commodities	- Different donors provide different supplies and services	- Short budget allocation on supplies	-Procurement decisions at the province level
	- Enough supplies were provided	- Conflict blocked supplies to the facilities	- Procurement systems to simplified and made efficient
		- Supply of fake medicines and documentation	- Strict monitoring
		- Allocation of budget for medicines not revised according to present needs	- Stringent quality checks
		- Absence of diagnostic facilities	
Monitoring and Reporting	- Developed SOPs	- Poor quality of data	- Promote E-Health
	- Encourage third party monitoring	- Preference of manual work over computer use	- Improve quality of data
	- EHIS system for reporting	- Capacity gap	- Do situational analysis before implementation
			-Data to be used for decision
Finances	- Donor dependent funding	- Insufficient funds	- To ensure sustainability of funding for existing programs
		- Delay in release of funds from the donors	
		- Poor practice of budget allocation and improper utilization of funds	

*CMW* Community Midwives, *SOPs* Standard Operating Procedure, *MnE* Monitoring and Evaluatio, *EHIS* Evaluation and Health Information System

Social, cultural and religious factors have also had their share on the uptake of health services especially contraceptives and immunization. The non-functional and poor quality healthcare facilities and structural in-competencies has affected provision of the quality of care and hence contributed to the low confidence of the people in approaching them [20, 21]. To overcome the shortage of health work force, the government should adapt collaborative policy which could devise ways for identification and retention of staff and also for capacity building and promotion of staff together with provision of incentives.

Although the implementation is decentralized through NGOs for delivery of health services at the provincial level; the decisions, control and monitoring is at the central level. This centralized system of decision making sometimes lacks proper budgeting and planning at provincial level and leads to lack of action at provincial level. Other studies has also suggested lack of strategic planning, inter-sectoral collaboration, poor information management system, weak policy making and monitoring systems [22]. Hence there is a need to ensure that the existing strong collaborative networks are fully channelized and strong monitoring and evaluation system put in place. There were also suggestions to explore internal revenues for financial support as Afghanistan cannot rely on external funding for a long time now and these challenges were reported by other studies [20, 23].

Our study though with limitations presents several implications for policy making in Afghanistan. The limitations included relying on the secondary quantitative data and obtaining robust and reliable data from a fragile state are challenging. Afghanistan has made progress in improving the health status but it now needs to scale up its efforts to make further inroads. For this it would require a holistic approach including sound infrastructure, strengthening of existing healthcare facilities, availability of trained staff (doctors, nurses, and vaccinators), capacity building of health workers and strong collaborations with a specific focus on improving quality of care and above all, the security situation should improve. The government should adopt a strategy with involving multiple stakeholders to identify high priority interventions which could vary between contexts within Afghanistan and explore innovative ways to ensure efficient delivery even during conflict. The gains in RMNCAH&N cannot be fully achieved unless involving related sectors including food security, economic development, poverty alleviation, improved education and enhanced infrastructure and communication network. Thus the ministry should take substantial steps in prioritization and planning of interventions, funds and resources to improve service delivery and for sustainable health outcomes.

## Conclusion

In the midst of conflict, progress in health system In Afghanistan has been hampered by weak stewardship, poor infrastructure, limited health workforce and poor monitoring and collaboration platforms, which has resulted in disrupted and fragmented health system. The government should adopt a collaborative approach to improve decision making and strategic planning for sustainable health outcomes.

## Supplementary information

**Additional file 1.** Interview Guide.

## Data Availability

The datasets used for analysis in this study are available from the corresponding author on reasonable request.

## Abbreviations

ANC: Antenatal Care
ARI: Acute Respiratory Infection
BCG: Bacillus Calmette–Guérin
BPHS: Basic Package for Healthcare Services
BRDs: Battle Related Deaths
CBHC: Community Based Health Care
CCI: Composite Coverage Index
CCT: Conditional Cash Transfer
CHW: Community Health Worker
CMWs: Community Midwives
CSO: Central Statistics Office
DHS: Demographic Health Survey
DHIS: District Health Information System
DPT3: Diphtheria-Tetanus-Pertussis
EHIS: Evaluation and Health Information System
EPI: Expanded Program on Immunization
EPHS: Essential Package of Hospital Services
EU: European Union
IDPs: Internally Displaced Persons
IYCF: Infant and Young Child Feeding
MICS: Multiple Indicator Cluster Survey
MoPH: Ministry of Public Health
NGOs: Non-Governmental Organization
OR: Odds Ratio
ORT: Oral Rehydration Therapy
PHC: Public Health Committee
PHCC: Provincial Health Coordination Committee
PNC: Postnatal Care
RMNCAH & N: Reproductive, Maternal, Newborn, Child, and Adolescent Health and Nutrition
SBAs: Skilled Birth Attendance
SOPs: Standard Operating Procedures
UCDP: Uppsala Conflict Data Programme
UN: United Nations
USAID: United States Agency for International Development
UNICEF: United Nations International Children’s Emergency Fund
WHO: World Health Organization
