# Supplementary material for: Impact of conflict on maternal and child health service delivery: a country case study of Afghanistan
Source: Confl Health. 2020 Jun 10;14:38. doi: 10.1186/s13031-020-00285-x (PMC7288441; doi:10.1186/s13031-020-00285-x)
Supplement: Supplementary file 1 — Additional file 1. Interview Guide. [file 13031_2020_285_MOESM1_ESM.docx]

**Interview Guide:**

I: Do you have any questions before we begin?

| **Generic Questions** | |
| --- | --- |
| 1 | **Can you describe your educational background?** |
| 2 | **Can you describe your work experience before entering your role as [role]?** |
| 3 | **Can you describe your work experience before entering your role?** |
| 4 | **We understand that working in Afghanistan can be quite difficult given the ongoing conflict. Can you elaborate on how the ongoing conflict has affected you and your work?** |
| Probe | Can you describe any personal precautions you have taken as a result of the conflict?  Can you describe (Probe for health care facility staff and UN agency staff only)   - How you and your team feel working in [current location]? |
| 5 | **Can you explain to me what your role as [position] with [Governing Entity/UN agency/NGO/facility] entails?** |
| Probes | How do you interact with   - Regional/provincial/district offices of Government/NGOs? - Head office?   Can you describe your role   - In the allocation of finances? - The allocation of resources and commodities? - In determining what geographic areas your organization works in? - Planning and delivering of RMNCAH&N interventions   Can you explain what geographic areas you are responsible for in your position as [role]?  How are you connected to the existing health system? (Probe for health care facility staff only)  Are you a part of an existing host country health system or do you consider yourself separate from the health services that already exist? |
| 6 | **We have been looking at [Governing Entity/NGO/facility]’s activities and have made a list of the services or programs that [Governing Entity/NGO/facility] delivers. Could I share this list with you? [Share list of services]. Is there anything missing from the list or anything listed that is no longer delivered?** |
| 7 | **So we have discussed [list services or interventions] as part of the services you provide. If you don’t mind I’m going to list a few other areas of health. Could you tell me if the government/NGO/facility provides any services or interventions related to these additional areas?** |
| Probe | Can you explain what considerations factored into the decision not to provide service? (Probe for NGOs and Government only) |
| 8 | **Can you briefly overview the current security situation in Afghanistan? (e.g. access, incidents)** |
| 9 | **Can you describe the impact of security constraints on your work? Can you give me some examples?** |
| 10 | **Can you describe how you manage security concerns?** |
| Probes | Were visas or special permissions required for personnel entrance into the country or for the deployment of commodities, medications, or equipment?   - From whom were permissions sought?   Were permissions required for the deployment of facilities or service providers?   - From whom were permissions sought?   To what extent were security considerations a basis for confining services to specific locations, such as IDP or refugee camp settings?  What role did [Governing Entity/NGO/UN agency] play in the location of health facilities or services?  With whom did the providers of services negotiate security?  Did security forces accompany the transport of personnel, commodities, or equipment to service locations?  What groups provided security for health facilities or providers of services?  Have security forces   - Influenced where or what services were provided? - Influenced which populations or patients were to be provided services? - Screened recipients of services before or at the service facility? - Interfered with the provision of services?   Have any humanitarian workers or facilities been victims of violence or received threats of violence? If so, from whom (if known)  Were non-standard fees or covert payments required to operate facilities?  Was there any coordination with any opposition forces/tribal councils/other groups/country-specific category to allow the delivery of your intervention? |
| 11 | **Can you explain how the sociocultural context (e.g. gender, caste, ethnicity etc.) influenced intervention selection/prioritization or delivery? Can you give me an example?** |
| Probes | Were there any subpopulations that were particularly hard to access? Can you describe this group?  Were there any special efforts to try to access this subpopulations? Can you give an example?  Were different delivery methods used for different services in the same region? Can you explain what is underlying this difference?  Did the sociocultural context affect one health domain in particular? Can you give me an example?  (context specific probes will need to be generated by each country team) |
| 12 | **Can you describe any other factors that affected the delivery or implementation of interventions?** |
| 13 | **Were there any changes to the services that were planned compared to those that were delivered?** |
| Probes | Can you describe how these services changed?  What factors influenced changes?  How did fluctuations in the level of funding affect service provision?  Can you describe the impact of the changing security context on the interventions delivered?  Were any formal or informal evaluations conducted? Did these indicate the need for a change in the interventions provided? Could you give me an example?  Were there any substantial influxes of displaced populations?   - Can you describe how this influx influenced intervention priorities? Can you give me an example? - Did [Governing Entity/NGO] receive additional funds to provide services to the influx of population?   Were there any epidemics while you were in the field?   - How did this shift priorities? Can you give me an example? - Did you receive additional funds to address the epidemic? - Can you describe how long the effects of epidemic were felt? - Can you describe what happened with health services once the epidemic was addressed?   Were there any natural disasters while you were in the field?   - How did this shift priorities? Can you give me an example? - Did you receive additional funds to address the impact of the natural disaster? - Can you describe how long the effects of the natural disaster were felt? - Can you describe what happened with health services once the situation with the natural disaster was resolved? |
| **Questions for NGOs, Government Staff and UN officials** | |
| 1 | **Just as a reminder, we’re focusing on the period between [country specific time period]. Could you describe what informed and/or continues to inform your decisions on which interventions to deliver?** |
| 2 | **Between [country specific time period], were any formal or informal needs assessments or surveys conducted to determine health priorities?** |
| 3 | **Can you describe how surveys, surveillance data, results of these assessments, or other data influenced the interventions [Governing Entity] provides?** |
| Probe | - If no assessments were done, why? (E.g. resources, political concerns, etc.) (Ask with Government official only) - What areas of health did these assessments focus on? - What type of data was collected in these assessments? - Can you describe the geographic areas where these assessments were conducted? - How was the decision made to conduct assessments in those specific geographic areas? - Can you describe the population assessed (e.g. IDPs, refugees, non-displaced etc.)? - How was the decision made to assess that specific population? - Can you describe how often these assessments were conducted? - Can you share more information about who conducted these assessments? - Would we be able to access these assessments? - Can you describe how any formal or informal evaluations of your programs influenced intervention priorities? |
| 4 | **Can you describe how scientific or academic literature influenced the interventions?** |
| 5 | **Can you describe how guidelines influenced the interventions being provided?** |
| 6 | **Can you describe how the cost-effectiveness of different interventions influenced the interventions?** |
| 7 | **During [country specific time period], can you describe how the availability of certain cadres of health workers influenced intervention priorities?** |
| Probes | Can you describe   - Any issues you had with retaining the workforce? How did you manage these issues? - The health worker recruitment and training process? Can you explain any issues you faced during recruitment? - Any interventions you had hoped to prioritize but couldn’t because of a lack of available workforce? - Any interventions that weren’t priorities but were delivered because of the available workforce?   Did workforce availability affect   - One health domain in particular? Can you give me an example? - One geographic area in particular? Can you give me an example |
| 8 | **Were there any concerns about the availability or shortages of commodities? Can you give me an example?** |
| 9 | **Can you explain how the availability of certain commodities influenced intervention prioritization?** |
| Probes | Can you describe   - Any adjustments that had to be made to the interventions provided as a result of commodity availability? Can you give me an example? - How stockpiles influenced intervention priorities?   Did [Governing Entity] receive any commodity donations for use in Afghanistan? Can you explain how these influenced intervention priorities?  Did commodity availability affect one health domain in particular? Can you give me an example? |
| 10 | **Focusing on the period between [country specific time period], can you explain how the level of funding received by [Governing Entity] affected what health interventions were prioritized?** |
| Probes | - Can you describe the primary sources of funding? - How did the sources of funding influence the types of services delivered? Were there any restrictions associated with this funding? - Were certain services deprioritized because of financial constraints? Can you give me an example? |
| 11 | **Can you describe any other elements or resources that influenced the prioritization of interventions?** |
| Probes | - Did you have any difficulties procuring these resources? How did you address these difficulties? - How did the shortages of [resource listed] influence the intervention? Were any adjustments made to the intervention due to [resource listed] shortages?   Were certain services deprioritized because of the availability of [resource mentioned]? Can you give me an example? |
| 12 | **Can you describe how you negotiate competing priorities? Can you give me an example?** |
| Probes | In what sense are priorities competing? (i.e. time, workforce, resources, capital etc.) |
| 13 | **In your capacity as [position] with [Governing Entity] have you participated in any cluster meetings? If so within which clusters?** |
| 14 | **Could you explain what decisions were made at these meetings? Can you give me examples?** |
| Probes | - Could you describe the extent to which you interacted with the groups at the cluster meeting after the meetings? - What are the differences between the services you provide and the services other members of the cluster group provide? |
| 15 | **Outside of the NGOs that attend the cluster meetings, as [role] with [Governing Entity] do you work with any other organizations?** |
| Probes | Does the government subcontract services to other organizations?   - What types of services are subcontracted? - Where are services subcontracted? - Can you explain why services are subcontracted? Can you give me an example? - Can you describe to what extent the subcontracted organization can influence the services or types of services delivered? |
| 16 | **Outside of your interactions during cluster meetings, can you list the UN agencies [Governing Entity] interacts with?** |
| 17 | **Can you explain to what extent your organization interacts with [UN agency]?** |
| 19 | **Within your organization whose responsibility is it to coordinate with the UN agency With whom do you interact at [UN agency]?** |
| Probe | Could you describe any specific government requirements that [ UN agency ] must adhere to if they want to work within the country? Can you give me an example? |
| **Specific Questions for NGO Staff** | |
| 1 | **Can you explain how your organization interacts with the government?** |
| 2 | **Whose responsibility is it to coordinate with government agencies? With whom do you interact with in the government?** |
| 3 | **What level of government agencies do you coordinate with?** |
| Probes | Can you describe how, if at all, your organization coordinates their service delivery with government services?  Could you describe any specific government requirements that [NGO] must adhere to if they want to work within the country? Can you give me an example?  Were there any activities that the government did not want you to report on? Could you give me an example? Can you explain this situation?  Can you describe any laws or regulations that restricted your activities within the country? How did you manage these restrictions? (e.g. abortion restrictions)  How did you manage constraints created by the government in a way that allowed [NGO] to do their work?  Does your organization receive any funding from the government? Can you elaborate on the details of this funding? |
| **Healthcare Staff Specific Questions** | |
| 1 | **Can you describe what interventions are delivered in which geographic areas?** |
| Probe | Are different interventions delivered in different geographic areas? Can you describe why and what underlies these differences? |
| 2 | **Were there any regional differences in delivery methods of the same intervention?** |
| Probes | Were different delivery methods used for   - Different interventions in the same region? Can you explain what is underlying this difference? - The same interventions in different regions? Can you explain what is underlying this difference?   Can you take me through the process through which you identify the best way to deliver interventions in that area? What do you consider?  Were different health workers used for   - Different interventions in the same region? Can you explain what is underlying this difference? - The same interventions in different regions? Can you explain what is underlying this difference?   Can you take me through the process through which you identify the best health worker to deliver interventions in that area? What do you consider? |
| 3 | **Can you describe obstacles you faced delivering this intervention? Can you give me examples?** |
| 4 | **Can you describe any innovative approaches that were used to overcome these obstacles?** |
| Probe | Did any of the obstacles you faced require revisiting the planned delivery method?  Were any other modes of delivery considered but eliminated as not feasible? Can you give me examples? |
| 5 | **Can you describe where recipients access your interventions?** |
| Probes | Can you describe the considerations that led to that location being identified as the place of delivery?  How did the use of this location assist in the delivery of the intervention?  How did the use of this location act as barrier to the delivery of the intervention? |
| 6 | **Were multiple interventions packaged together?** |
| 7 | **Who accessed your interventions? Can you describe this group? (Gender? Age? Educational status? Refugee and/or IDPs? Camp residents and/or dispersed population? Etc.)** |
| Probes | Who were the intended recipients of interventions?  Were there any regional variations in the type of recipient who accessed interventions?  Were there differences between intended recipients and actual recipients? If so, why do you think that is?  Were there any groups that this delivery method did not work well for? Can you describe this group?  Were there any special efforts to try to access this group? Can you give an example? |
